# Recent Advances in the Characterization of Genetic Factors Involved in Human Susceptibility to Infection by Schistosomiasis

**DOI:** 10.2174/138920208785133262

**Published:** 2008-08

**Authors:** Amandine Isnard, Christophe Chevillard

**Affiliations:** 1 INSERM, U906, Marseille, France, Faculty of Medicine La Timone, University of Aix-Marseille, France; 2 Laboratory of Parasitology Mycology, Faculty of Medicine La Timone, University of Aix-Marseille, France

## Abstract

Human resistance to infection by schistosomes is associated to a strong Th2 immune. However a persistent Th2 response can cause severe kidney and liver disease in human. In this review, we mainly focused on the control of infection levels caused by schistosomes. Several experimental models allowed us to better understand the immunological mechanisms of the host against schistosome infection. High IgE and eosinophil levels are associated with resistance to infection by schistosomes and this effect is counterbalanced by IgG4. IgE and eosinophils are highly dependent on IL-4, IL-13, and Il-5, which are three main Th2 cytokines. We also examined the genetic factors involved in human susceptibility to infection by schistosomiasis. Infection levels are mainly regulated by a major locus SM1, in 5q31-q33 region, which contains the genes encoding for the IL-4, IL-13, and Il-5 cytokines. An association between an *IL13* polymorphism, rs1800925, and infection levels has been shown. This polymorphism synergistically acts with another polymorphism (rs324013) in the *STAT6* gene, encoding for the signal transducer of the IL13 pathway. This pathway has also been involved in atopic disorders. As helminthiasis, atopy is the result of aberrant Th2 cytokine response to allergens, with an increased production of IL-4, IL-13, Il-9 and Il-5, with high amounts of allergen-specific and total IgE and eosinophilia. However, the Th2 immune response is protective in helminthiasis but aggravating in atopic disorders. Several studies reported interplay between helminthic infections and allergic reactions. The different results are discussed here.

## INTRODUCTION

Infectious diseases are responsible for 45% of death in developing countries (25% of deaths throughout the world). Schistosomiasis (or bilharziasis) is the second leading parasitic disease behind malaria and remains a major public health problem. Approximately 300 million people are exposed to *Schistosoma* in 74 countries worldwide with a concentration in Asia, Africa, and South America [1]. Each year 280,000 people die of this disease [2].

Schistosomiasis is caused by the digenetic trematode *Schistosoma*. Three main species are pathogenic for humans, each one causing a different clinical presentation of the disease. *Schistosoma mansoni* is responsible for hepatic bilharziasis, *Schistosoma haematobium* leads to urinary bilharziasis, and *Schistosoma japonicum* is responsible for hepato-intestinal bilharziasis. Moreover, these three forms present different geographical distribution. Infection of *Schistosoma mansoni* (*S.m.*) is found in South America (Brazil, Venezuela, and Surinam), Africa and the Middle East. Infection of *Schistosoma haematobium* (*S.h.*) occurs mainly in Africa and in the Middle East, while *Schistosoma japonicum* (*S.j.*) is found in the Far East, mostly in China and the Philippines.

## PARASITE CYCLE 

Schistosomes have a typical trematode vertebrate-invertebrate lifecycle Fig. (**1**). Sexual reproduction of *Schistosoma* occurs in the definitive host (humans) and asexual reproduction occurs in the intermediate hosts (snails). These latter hosts are different depending on the *Schistosoma species*. Thus, *S.m. *penetrates into *Biomphalaria spp*, *S.h. *penetrates into *Bulinus spp*. and* S.j.* infects *Oncomelania spp*.

Fertilized schistosome eggs, delivered in fresh water *via* excrements of infected subjects, hatch after ~10 days and release ciliated motile miracidia. Miracidia infect the intermediate hosts by penetrating the foot of the snail. They transform into primary sporocysts, which then begin division into secondary sporocysts. The secondary sporocysts migrate to liver and pancreas and divide again into thousands of larvae termed cercariae, which are capable of infecting mammals. Four to six weeks later, mature cercariae emerge daily from snails in a circadian rhythm, depending on ambient temperature and light.

People are infected by contact with water used in normal daily activities (such as washing, wading, fishing, rice cultivation…). Young cercariae directly penetrate the body surface by attaching to human skin and by secreting enzymes that break down the skin proteins. During this process they loose their tail and transform into schistosomules. The schistosomules may remain trapped into the skin for 2 days before entering the vasculature and migrating to the lungs. There, parasites undergo further developmental changes (8-15 days after infection) and move to the portal circulation and finally to the liver, where maturation and mating between males and females occur. 

At this step, the female worm resides in the gynaecophoric channel of the male. The worm pairs move against the flow of blood into their final niche. Adults *S.m.* and *S.j.* thus reside in the venous mesenteric plexus, whereas adults *S.h. *reside in the venules of the urinary bladder and ureters. Adult worms measure 1-2cm in length and generally live 3-5 years in the human body, but they may persist up to 20 years. Female worms release eggs (300-3000 eggs/days), which measure 70-150µm in length, within the vasculature. Usually, these eggs cross the endothelium and basement membrane of the vein and traverse the intervening tissue, such as basement membrane and epithelium of the intestine (*S.m. and S.j.*) or bladder (*S.h.*) in order to be eliminated in urine or faeces Fig. (**1**).

## PATHOPHYSIOLOGY

The pathophysiology of *Schistosoma* infection can be divided into three stages: First, the cutaneous stage is discreet and consists of movement of cercariae through the skin. Penetration and migration of cercariae are facilitated by proteolytic enzymes secreted from cephalic glands capable of digesting epidermal keratin [3]. Skin reactions (itching) may develop within a few hours after infection. A rash may appear up to one week later.

Then, acute schistosomiasis is a clinical syndrome often seen in non immune individuals (tourists, immigrants, or the indigenous population), who have been exposed in an endemic area to a primary infection by cercariae. This syndrome is also called Katayama fever [4, 5]. The acute intestinal symptoms due to *S.m. *infection appear when adult worm pairs release eggs in the tissues. Symptoms include diarrhea, which may start suddenly 40-55 days after the primary infection and may last for 6-12 months.

Finally, chronic manifestation of *Schistosoma* infection occurs as a consequence of many years of progressive injury, resulting from chronic egg deposition in the tissues [6]. In *S.m. *infection, eggs are swept into the portal circulation and can be trapped within the intestinal wall. This phenomenon leads to the chronic intestinal disease, during which patients complain of abdominal pain. In the *S.j.* infection, eggs are likely deposited along the small portal veins of the peripheral part of the liver. This is probably because eggs of *S.j.* are smaller (70-100µm) than those of *S.m.* (110-170µm). During *S.h.* infection, the passage of eggs across the bladder wall causes damage to this organ.

Eggs trapped in liver are surrounded by the granulomatous inflammatory response of the host (immune response benefit to the host if well-controlled) [7-9]. Hepatomegaly reflects the presence of granuloma and occurs early in the evolution of the chronic disease. The intensity and the duration of infection determine the amount of antigens released and the severity of the disease. The granuloma that is regulated by a variety of cytokines and mediators [10, 11] destroys the eggs but this is sometimes accompanied by excessive fibrous tissue deposition, which concentrates in the periportal spaces [12] and leads to the development of periportal fibrosis (Symmers’ fibrosis) [13]. Associated with this phenomenon are a periportal hypertension [14], the development of ascites and portal-systemic venous shunts, which can rupture and lead to life-threatening bleeding.

The most serious effects of infection with *S.h.* are kidney dysfunctions, bladder cancer and genital schistosomiasis, in which eggs pass through the cervix in women or into the testes in men, and which can lead to sterility. 

## MULTIPLE FACTORS ARE INVOLVED IN HUMAN RESISTANCE TO SCHISTOSOMIASIS

Schistosomiasis is a multifactorial disease involving environmental, behavioral, parasitic, vector and host factors [15]. For instance, the number of parasites in infected water varies depending on the studied area and is thus one of the main environmental factors. For a long time we have thought that the most exposed individuals were also the most infected, as determined by fecal egg counts. However, a study carried out on a fisherman population from Uganda showed that adults, who were more exposed than children because of their occupation as fishermen, were less infected than the children [16]. Actually, age appeared as an essential host factor. Indeed, children younger than 15 years old are generally more susceptible to infection than adults [17-20]. Thus, an acquired protective immunity would appear gradually according to the age and exposure by an individual [21]. In these conditions an early and repeated exposition to infection would lead to an earlier establishment of protection.

Sex is another host factor implicated in the control of infection, with higher infection levels in males than in females, which could be explained by reduced infected water contact after puberty for women or/and by hormonal factors. Moreover in endemic areas of schistosomiasis, it was observed that individuals with the highest infection levels were grouped within certain families, rather than randomly distributed and that certain subjects were clearly predisposed to reinfections [15]. This suggested that human resistance to infection by *Schistosoma* may also depend on the effects of some genetic factors [15, 22-24].

## TWO MAJOR LOCI CONTROL HUMAN SUSCEPTIBILITY TO SCHISTOSOMIASIS

In 1991, Dessein *et al*. performed segregation analysis, the first step to determine the mode of inheritance of a given trait from family data, on 20 Brazilian pedigrees (269 individuals), issued from an endemic area of *S.m.*, in the aim to investigate whether a major gene controls human susceptibility/resistance to infection by *Schistosoma* [25]. Two different models of analysis were used: the unified mixed model and the regressive model, and relevant factors for schistosomiasis, such as water contact, sex and age were taken into account. Results showed that the best model to explain the inheritance of the control of infection by *Schistosoma* was the existence of a codominant major gene. This gene accounts for 66% of the infection intensity variances residual from covariate effects. The deleterious allele occurs with a frequency of 0.16. This frequency correlates with 3% of the population homozygous for the allele and thus predisposed to high infections, 27% of the population heterozygous with an intermediate level of infection, and 70% of the population resistant. These findings provide a genetic basis for earlier observations on the lower resistance and the predisposition to reinfection of some individuals.

Marquet *et al*. (1996) showed that variations of infection levels with age cannot be fully explained by changes in water contact [26]. Indeed infection levels adjusted for water contact increased markedly between the time of first contact (~ age 2 to 3) and puberty, and then declined during adolescence, which correlates with the view of an assumption of gradual acquisition of immunity during childhood.

To localize the major gene involved in the control of infection by *Schistosoma*, referred as SM1, the authors also carried out a genome-wide study on 142 Brazilian subjects belonging to 11 informative families (2 large pedigrees, 5 smaller pedigrees and 4 nuclear families). In a primary map (246 markers; interval of 15cM between adjacent markers), two adjacent markers on chromosome 5q31-33, D5S393 and D5S410, provided lod scores greater than 1.9, indicating a suggestive linkage [26]. Therefore, 11 additional markers in this region were analysed. Significant evidence of linkage (Z>3.3) was obtained with 2 proximal markers: D5S636 (Zmax=+4.74, θ=0.07) and the Colony Stimulating Factor-1 Receptor (CSF1R) (Zmax=+4.52, θ=0.04). Refined multipoint analysis of the region confirmed that the most likely location of SM1 was in close proximity to CSF1R, with a Zmax=+5.45 observed between CSF1R and D5S636 [26]. This result was confirmed later in an independent study done in a Senegalese population living in an endemic area for *S.m.* [27].

This region contains several candidate genes involved in the regulation of the immune response to pathogens, such as the genes encoding for the interleukins 4 (IL-4), IL-5, IL-9, and IL-13 [28], all known to induce an anti-inflammatory Th2 response. The region also contains the gene encoding for the IL12p40 subunit common to the proinflammatory cytokines IL12 and IL23 [29]; the Interferon Regulatory Factor 1 (IRF1), which encodes a transcriptional activator of interferon-α (IFN-α), interferon-β (IFN-β) and other IFN-inducible genes [30]; and finally the Colony-Stimulating Factor 1 Receptor (CSF1R) gene [31]. Furthermore, the 5q31-q33 region has already been associated with the regulation of IgE levels [32], to familial eosinophilia [33], asthma [34], *Plasmodium falciparum* blood infection levels [35], and more recently, to inflammatory bowel diseases [36].

Marquet *et al*. found that four additional markers in three other regions provided interesting lod-score values above 0.83: 1p22.2 with D1S216 (Zmax=+0.91, θ=0.20), 21q22-qter with D21S1259 (Zmax=+1.09, θ=0.19) and the two adjacent markers D7S483 (Zmax=+0.91, θ=0.20) and D7S550 (Zmax=+1.02, θ=0.22) in the 7q36 region [26, 37]. Although these values are not significant in the context of a genome wide search, these regions contain some interesting candidate genes. The IL12 receptor β2 chain gene is located close to the 1p22.2 region [38], and plays an important role in the regulation of the Th1-type immune response. The interferon-α receptor (IFN-αR) and interferon β receptor (IFN-βR) genes, both inhibitors of IL-12 and INF-γ, are located in 21q22-qter [39]. Finally, the beta T cell receptor (TCRβ) gene maps closed to the 7q36 region [40].

Dessein *et al*. (1999) showed that genetic factors were involved in the control of severe disease caused by *Schistosoma *[14, 41]. This work was carried out in a Sudanese population free of treatment and living in a region endemic for *S.m.*. Fibrosis was evaluated by ultrasound, liver size, peripheral portal vein branches (PPBs), spleen size, and splenic and portal vein diameter (PVD). Periportal fibrosis was graded 0 to 3 as suggested by the Niamey working Group.

Several observations have suggested that inherited factors might play a role in advanced fibrosis in this Sudanese population, such as elevated frequency of advanced fibrosis in certain families, despite similar epidemiological conditions for the whole study population and that fibrosis grades correlated only between parents and children but not among spouses. Dessein *et al*. performed a segregation analysis on 781 subjects (361 males, 420 females) belonging to 65 pedigrees [14]. The hypothesis of no familial dependence was rejected, and sib-sib dependence was not significant. The results showed that a codominant major gene referred to as SM2 accounts for the familial distribution of the phenotype defined as hepatic fibrosis and portal hypertension in schistosomiasis. The frequency of the deleterious allele A was estimated as 0.162. Therefore, the proportions of AA, Aa and aa subjects were 0.03, 0.27and 0.70 respectively.

To localize SM2, the authors genotyped all the informative families with multiple cases of severe fibrosis (8 families including 112 individuals) and linkage analysis was done in four candidate regions: (1) the 5q31-q33 region were SM1 and several candidate genes are localized; (2) the HLA-TNF region (6p21) containing the HLA locus and the genes coding for TNF-α and TNF-β; (3) the 12q15 region including the gene coding for IFNγ and a gene controlling total serum IgE levels; (4) the 6q22-q23 region containing the IFNγR1 gene. No significant results were found in the 5q31-q33, the 6p21 and the 12q15 regions. However significant Zmax values were observed in the 6q22-q23 region, with both D6S310 (Zmax=2.81, θ=0.0) and the FA1 intragenic marker (Zmax=1.80, θ=0.0) [41].

## IMMUNE RESPONSE ASSOCIATED TO INFECTION

In the murine model, several reports showed that a downregulation of Th1 cytokine production and an induction of a Th2 response lead to a high IgE levels. Moreover, high numbers of peripheral eosinophils were associated with a poor clinical outcome [42].

In humans, analysis of the immune response of individuals with different levels of resistance were done to understand the principal mechanisms of resistance and to identify their targets on the parasite. Rihet *et al*. (1991) evaluated IgE-dependent immunity in subjects with high resistance to infection. They observed that IgE levels remained relatively steady during the whole study, while subjects experienced large variations in infection intensities, indicating that aborted or low infections are sufficient to maintain high IgE levels. They also showed that IgE bound to a large variety of *S.m.* antigens. Some of them were on the outer part of the schistosomule membrane, suggesting that these parasitic regions were putative targets of IgE-dependent cell-mediated cytotoxicity against the invading larvae. Finally, they demonstrated that IgE levels were higher in the most resistant subjects than in the less resistant ones [43]. All these observations are consistent with the fact that IgE play a significant role in human immune defenses against *S.m.* infections. It has also been reported that human eosinophils, purified from patients infected with *S.m.*, expressed three different types of IgE receptors: the high affinity Fc-ε receptor (Fc-ε RI), the low-affinity Fc-ε receptor (Fc-ε RII/CD23) and Mac-2/ εBP [44-46].

In the aim to resolve contradictory findings concerning acquired immunity between mice and humans, Nyindo *et al*. (1999) used baboons as an experimental model [47]. Nonhuman primates and particularly the olive baboon (*Papio cynocephalus anubis*) have several advantages over rodents. Indeed, in East Africa, *S.m.* infects wild populations of olive baboons and can sustain transmission removed from human contacts. Baboons are highly susceptible to experimental infections as well. The proportion of penetrating cercariae that mature to adult worms often exceeds 90%, whereas cercarial infectivity in mice rarely surpasses 50%. Natural infection as well as immunization with irradiated cercariae or recombinant antigens of schistosomes, produce partial resistance to challenge infection in baboons. The pathologies in baboons closely resemble human infection in that the baboons acquire immediate hypersensitivity responses to schistosomes’ antigens. In an initial experiment, animals were exposed to single or multiple infections and were subsequently cured with praziquantel prior to challenge with another infection. Singly and multiply infected animals mounted reductions in worm burden. In a second experiment, animals were inoculated with *S.m. *ova and recombinant human interleukin 12. This produced a reduction in adult worm burden after challenge. Parasite-specific IgG, IgE, IgM, and peripheral blood cytokine production were evaluated. The only immune correlate of protection in both experiments was levels of soluble adult worm antigen (SWAP)-specific IgE in serum at the time of challenge infection and/or 6 weeks later [47]. Thus, in baboons and not in mice, adult worm-specific IgE is uniquely associated with acquired immunity to *S.m. *infection.

Finally the best and the most used experimental model of infection by *Schistosoma* was shown to be the rat. The rat is a semi-permissive host to *S.m.* infection, as it can reject worms between 3 and 4 weeks after a primary infection, and develop immunity to reinfections [48, 49]. In this experimental model, humoral immunity plays a major role, whereas the cell-mediated immunity does not [48, 49]. Thus, the rat was used to identify the antibody isotypes involved in the antibody-dependant cellular cytotoxicity (ADCC). First, it was shown that the anaphylactic antibodies IgG2a and IgE enabled *in vivo* killing schistosomules by eosinophils. This cytotoxicity was enhanced by mast cells, which release soluble factors that activate eosinophils [50]. Then, the use of monoclonal antibody isotypes IgG2a and IgE, as well as eosinophils primed with cytophilic IgE, confirmed these observations [51, 52]. Among the monoclonal antibodies produced, IgG2c was not cytotoxic but was able to inhibit the *in vitro* effects of IgG2a, by blocking eosinophil degranulation and cell-mediated cytotoxicity [53, 54]. Moreover, Dombrowicz *et al*. (2000) demonstrated that in rats, as in humans, eosinophils and macrophages expressed a functional alpha gamma 2 trimeric Fc-ε RI and that these two cell types could induce IgE-mediated, Fc-ε RI-dependent cellular cytotoxicity toward schistosomules [55].

A similar isotypic regulation happens in human infection. First, Khalife *et al*. (1986) showed that high levels of the IgM isotype were associated with schistosomiasis reinfection in children classified as susceptible to posttreatment reinfection. This was determined to be due to IgM blockade of eosinophil-dependent cytotoxicity [56]. Likewise, Hagan *et al*. (1991) demonstrated a correlation between IgG4 isotypes and a strong susceptibility to reinfections [57]. IgG4 antibodies block eosinophily-mediated killing that is aided by IgG effector antibodies, such as IgG1 and IgG3. In addition, IgG2 antibodies mediate activated eosinophil effector function but block normal eosinophil function. These results were confirmed by Demeure *et al. *(1993) who found a positive association for IgE and negative associations for IgG2 and IgG4 with resistance to reinfection after chemotherapy. The opposing effects of IgE and IgG4 were undissociable in the analysis, suggesting that these isotypes antagonize each other in protection [58].

As IgE and eosinophils are highly dependent on IL-4, Il-5 and IL-13, it was thought that protection against infection by *Schistosoma* would involve a Th2 immune response. Therefore Couissinier-Paris *et al*. (1995) asked whether human resistance to *S.m.* was associated with a particular T helper subset. They isolated twenty-eight CD3^+^, CD4^+^, CD8^-^ parasite-specific T cell clones from three adults with high degree of resistance to infection by *S.m.*. The lymphokine secretion profiles of these clones were determined and compared to those of 21 CD3^+^, CD4^+^, CD8^-^ clones with unknown specificity, established from these same subjects in the same cloning experiment. They found that the parasite-specific T cell clones isolated from adults resistant to *S.m.* produced large amounts of IL-4 and IFN-γ and belong to the Th0 subset. However they produced more IL-4 than IFN-γ (Th0/2). They then compared parasite-specific T cell clones from the resistant subjects to specific T cell clones from a sensitized adult living in a nonendemic area: they showed that these latter clones were also Th0 but produced more IFN-γ than IL-4 (Th0/1) [59].

As highlighted above, human susceptibility to *S.m.* infections is controlled by the SM1 locus in the 5q31-q33 region. This genetic region encodes several cytokines that regulate the differentiation of Th1 and Th2 lymphocytes. Rodrigues *et al*. (1999) reported that they had performed a clonal analysis of CD4^+^ T lymphocytes in resistant and susceptible subjects to evaluate whether this genetic control is acting on Th1/Th2 pathways. The subjects evaluated were either homozygous for the allele that determines resistance to infection or were homozygous for the allele that determines high rates of infection. Of 121 CD4^+^ T cell clones (TCC) from three susceptible and three resistant subjects, 68 were parasite-specific (spTCC). The spTCC derived from susceptible subjects (33 STCC) produced 10- to 1000-fold less IL-4 and IL-5 than TCC from resistant subjects (25 RTCC). However clones from both patient groups produced the same amount of IFN-γ. Parasite-specific STCC were Th1 or Th0/1, whereas RTCC were Th2 or Th0/2. These results indicated that the SM1 locus controls the differentiation of Th2 lymphocytes [60].

## IL13 GENE IS ESSENTIAL TO THE CONTROL OF INFECTION LEVELS IN SCHISTOSOMIASIS

Infection levels by *Schistosoma* are controlled by a major locus on chromosome 5q31-q33 containing the *IL4, IL5* and *IL13* genes related to the Th2 immune response [26]. As previous studies have shown that sterile immunity in schistosomiasis is dependent on IgE levels, eosinophils and on the Th1/Th2 balance, our laboratory decided to test whether any allelic variants in the *IL4, IL5* and *IL13* genes would predispose individuals to high infection levels by *Schistosoma *[61]. A genetic association study was performed on a Malian population localized in two villages, Ségué and Boul, where *S.h.* is endemic (693 subjects in Ségué and 148 in Boul). *Schistosoma* infection levels were evaluated either by counting eggs in urine or by circulating worm antigens (CAA) in serum. We focused on areas of the promoter regions of *IL4, IL5* and *IL13* genes that contain transcriptional regulatory elements. Genotyping of the polymorphisms by restriction enzyme analysis or by primer extension and denaturing high-performance liquid chromatography analysis defined one polymorphism in the *IL4* promoter (IL4-590C/T), one in the *IL5* promoter (IL5-202G/A) and three polymorphisms in the *IL13* promoter (IL13-1258A/G, IL13-1055C/T (rs1800925) and IL13-591A/G (rs2069743)). We also added the functional polymorphism in asthma, IL13Arg130Gln, to the analysis [62].

Initially, a family-based study was performed to determine if one of the tested allele was preferentially transmitted to highly infected children [61]. In this case, the associated polymorphism would be located close to the susceptibility gene. A trend for an association for the polymorphism rs1800925 in an additive model was observed (p=0.05). In a recessive model, rs2069743 also shown an association (p=0.003). These results suggested that alleles rs1800925C and rs2069743A may predict an increased risk of infection Fig. (**2A**). A multivariate analysis was then performed between IL13 polymorphisms and high infection levels, taking into account age, gender and the village of origin. Age (p<10^-3^), gender (p<10^-3^), village (p<10^-3^) and rs1800925 (p=0.04) were all significantly associated with infection levels, while rs2069743 was rejected by the analysis. This association was even stronger when the analysis was limited to the highest infected village Boul (p=0.002). Genotypes rs1800925C/C and C/T were associated with the highest infection levels, whereas the T/T genotype was associated with the lowest infection levels Fig. (**2C**).

However the rs1800925 genotype effect could be due to an effect as well on egg excretion as on the schistosome larvae. To determine which of these hypotheses was correct, an association between rs1800925 genotypes and the high CAA concentrations was evaluated. The rs1800925T/T genotype was associated with lower CAA concentrations than the two other genotypes. To further test these results, a multivariate analysis was performed, including other variables known to affect CAA levels. A trend (p=0.01) was found again for an association between rs1800925 genotypes and CAA levels when taking into account age (p<10^-3^) and the village of origin (p<10^-3^). The other variables were excluded by the analysis. Once again, when the study was limited to Boul, the association became stronger (p=0.02). Thus, the rs1800925 genotypes are also associated with worm load, with the rs1800925T/T subjects having the lowest worm load. Collectively, these results suggest that IL-13 may be involved in protective immunity against both larvae and eggs.

The rs1800925 polymorphism has been demonstrated to be functional in a study dealing with allergic asthma [63]. The authors showed that the rs1800925T/T genotype was more frequent in allergic asthma patients (n=101) than in non-atopic controls (n=107) (p=0.002, OR=7.8). T cell IL-13 production is usually inhibited by a calcium-inducing signal, which can be reversed by CsA. They also showed that the T/T genotype was associated with a decreased relative inhibition of IL-13 production compared to the C/C (p=0.0016) or the C/T (p=0.0002) genotypes, as well in the patient group and in the control group. Finally they completed these observations by demonstrating that the change of the rs1800925C to the T allele increases the binding of transcription factors, such as NF-AT. Therefore, in the absence of CsA, known to prevent nuclear translocation of NF-AT through inhibition of the calcium-dependent phosphatase calcineurin, calcineurin activates NF-AT by dephosphorylation resulting in NF-AT translocation to the nucleus where it binds to specific DNA sequences that control gene transcription. The authors speculated that the binding of NF-AT to the IL-13 promoter inhibits IL-13 production.

A more recent study [64] reported on the analysis of the functional impact of this polymorphism according to the cell type. The authors showed that the T allele enhanced the IL13 promoter activity in primary human and murine CD4+ Th2 cells. The presence of this allele correlates with a Ying-Yang 1 factor (YY1) binding site but this site overlaps a STAT6 binding site which seems to inhibit the expression of the IL-13. Thus, binding of YY1 would inhibit the activity of STAT6, which would lead to increased IL-13 expression. They also confirmed that the rs1800925T/T homozygotes had a greater expression of IL-13, indicating that this *in vivo* genotype should be associated with susceptibility to allergic inflammation. Interestingly, in non-polarized CD4+ T cells, the rs1800925T allele caused the fixation of NF-AT2 in addition to those already reported above resulting in an opposite transcriptional effect.

## STAT6 POLYMORPHISM HAS SYNERGIC A EFFECT WITH IL13 POLYMORPHISM

The bronchial pathology in asthma and the protective host response to helminth include main elements of Th2 immune activity, which lead to increased mucosal eosinophil activity, mucus hypersecretion, and muscle hyperactivity. However, in the bronchus, these features correspond to the symptoms of chronic asthma, while in the gut these immune mechanisms promote the expulsion of helminths. Common upregulating genetic variants of Th2 immune signaling are risk factors for asthma and it was hypothesized that they may confer a counteradvantage in promoting protective Th2 immune response against helminth infection.

Peisong *et al. *(2004) tested whether Th2 risk polymorphisms for asthma were also associated to protection against the helminth worm *Ascaris lumbricoides*, in a Chinese population of 614 schoolchildren (11-15 years old). By logistic regression, they found a major association between a common genetic variant of the 3’-UTR regulatory elements (+4219G/A) of the Signal Transducer and Activator of Transcription 6 (STAT6) and egg counts (p=0.0002). Linear regression after log transformation of egg counts confirmed a highly significant association with this *STAT6* variant (p=0.001). They thus demonstrated that an asthma-associated risk polymorphism in *STAT6*, one of the main transcription factors in the Th2 signaling pathway, predicts increased resistance to ascaris worm infection [65].

These observations suggested that several genes encoding the main components of the Th2 pathway could have significant effects on *Schistosoma* infection. Recently, we decided to test whether any allelic variants of the major Th2 genes were associated with the control of *S.h.* infection levels. These studies were performed on samples from the same Malian population. We analyzed 18 polymorphisms contained in *IL4* (a major Th2 cytokine), *IL4RA* (the common receptor chain for IL-4 and IL-13), *IL13RA1*, *IL13RA2* (both the receptor chains for IL-13), *STAT6* (the signal transducer activated by the IL-4/IL-13 pathway) and *GATA3* (a major factor in determining Th1/Th2 responses) genes by multivariate analysis (linear logistic regression) [66].

We found a trend (p=0.04) of association for only one polymorphism, rs324013, localized in the *STAT6* gene promoter, taking into account age (p<10^-3^) and gender (p<10^-3^). The genotype rs324013C/T was found in subjects with higher levels of infection than subjects carrying the C/C or T/T genotypes Fig. (**2B**). This effect was more significant in the village with the highest levels of infection, (Boul Fig. (**2D**)), and in subjects under 20 years old. A separate linear regression analysis for subjects under 20 years old and from Boul showed a significant association between rs324013C/T and the highest infection levels (p=0.005).

In order to characterize the involvement of IL13 and STAT6 polymorphisms, a new multivariate analysis was done. Gender (p<10^-3^), rs324013 (p=0.017) and rs1800925 (p=0.027) were all associated with infection levels. The additive effect of these two polymorphisms was illustrated by subjects from Boul with the genotypes rs1800925C/C and rs324013C/T having higher levels of infection than subjects carrying the other genotypes Fig. (**2E-F**). A linear regression analysis confirmed the association of these two genotype combinations with infection levels (p=0.011).

We then checked whether these two SNPs directly affected infection levels or were correlated with the causative polymorphisms. We thus defined the correlation groups, based on r squared values, in the 5q31 region surrounding the *IL13* gene and the 12q13 region surrounding the *STAT6* gene. We found that rs1800925 (0<r^2^<0.22) and rs324013 (0.03<r^2^<0.24) were not strongly correlated with any other tested markers, indicating that they are likely to be the causative polymorphisms involved in the control of infection by *Schistosoma*. Finally, as both polymorphisms are localized in the promoter of their own gene, we performed gel shift assay to test if these allelic variants modify the binding of transcription factors depending on the present allele. We confirmed that rs1800925 changes the binding of transcription factors in PHA-stimulated PBMCs nuclear extracts and in CD4^+^ T cells nuclear extracts. We also demonstrated a differential binding for rs324013 in PHA-stimulated PBMCs, confirming thus that these two polymorphisms are functional. All these data are in agreements with the properties of IL-13 Fig. (**3**) and STAT6 proteins Fig. (**4**).

## HELMINTH, ASTHMA AND ALLERGY

Helminthiases and atopic disorders are closely linked. Indeed, both helminthic parasites and many allergens trigger highly polarized Th2-type immune responses. However, while in helminth infections this kind of immune response often leads to the worm expulsion or sequestration, in atopy it leads to severe allergic phenotypes. Several groups studied the effects of helminthic infections in allergic patients or the allergic reactions in patients infected by helminths.

Lynch *et al*. evaluated the influence of atopy on the antiparasite response in two groups of Venezuelan children, living in an endemic region for the intestinal helminth *Ascaris lumbricoides*, but differing greatly on their level of atopy, depending on whether they live on an island or in a mainland population [67]. They found that the intensity of the parasitic infection was considerably higher in the nonatopic mainland children than in the atopic island group, but with a ratio of anti-Ascaris specific to total IgE levels significantly more elevated in the atopic island subjects than in the nonatopic children. The atopic children seemed thus to have an intrinsic capacity to favor specific over polyclonal IgE responses concordant with an enhanced protective response against helminthic parasites.

Araujo *et al*. (2000) showed that in a region endemic of schistosomiasis (*S.m.*), there were a higher proportion of allergic individuals in the uninfected group than in the infected one, with a risk of developing atopy higher in the uninfected group [68]. They also found that the total and *S.m.* specific IgE levels were higher in infected group, whereas the aeroallergen-specific IgE were higher in the uninfected group. Therefore they demonstrated a strong and significant inverse association between the immediate skin test response to common aeroallergens and infection by *S.m..* Van den Biggelaar *et al*. (2000) confirmed these results in a study performed on a Gabonese population in which they found that children with urinary schistosomiasis (*S.h.*) had a lower prevalence of a positive skin reaction to house-dust mite than those uninfected [69]. Moreover, Medeiros *et al*. (2003) evaluated the influence on *S.m.* on the course of asthma [70]. They thus compared 3 groups of Brazilian asthmatic patients, issued either from a rural area endemic for *S.m.* or from non-endemic rural or slum areas, and matched according to their age and gender. Once again, they found a significantly milder form of asthma in the infected subjects. It is noteworthy that the protective effect of schistosomes on atopic disorders can happen in genetically different people from 2 continents (Africa and South-America) and that, at least, two species of *Schistosoma* can be involved.

Afterwards, several studies on children who had various chronic intestinal helminth infections in several developing countries, such as Ethiopia [71], Gambia [72], Taiwan [73] and Ecuador [74], confirmed the link between the Th2 immune responses, the chronic intestinal helminth infections and the reduced prevalence of allergic disorders. Furthermore it has been shown in various studies that the use of anthelminthics in infected subjects to remove worms increased their atopic reactivity to different allergens within ~2 years after treatment, raising the fact that the presence of helminths could actively suppress allergic reactions. However, the link between asthma/atopy and helminth infections was not clearly found in three other studies [75-77] indicating that this disease relationship is highly complex.

Finally, the recent advances in our knowledge of how genetic factors control the infection in schistosomiasis allow us to better understand the pathologic mechanisms of the disease. Thanks to these findings, we will be able to define new targets and hopefully to develop more efficient methods for diagnostics, therapies and vaccine.

## Figures and Tables

**Fig. (1):**
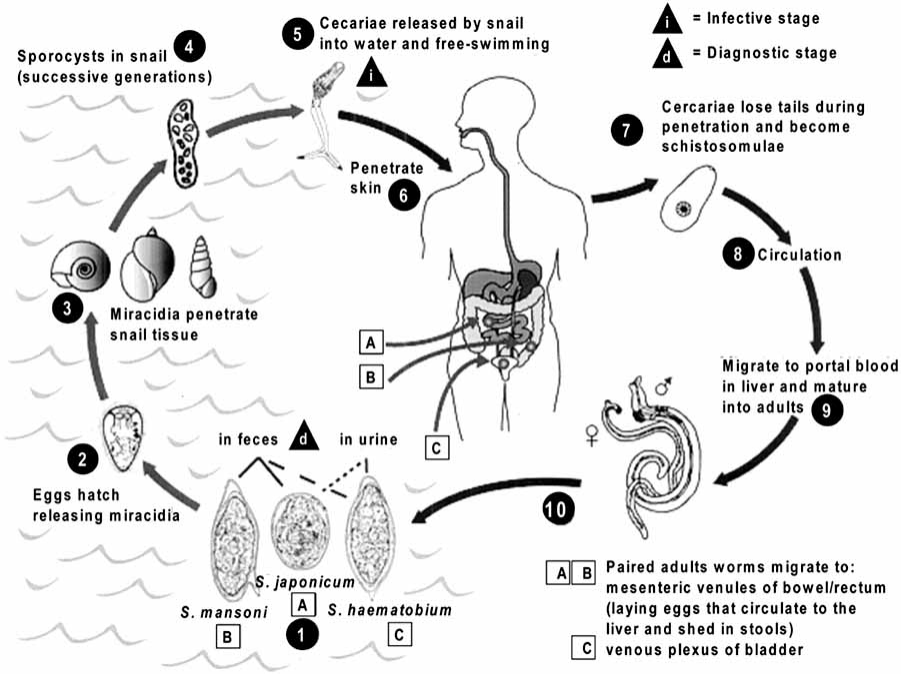
Parasitic life cycle of *Schistosoma mansoni, S. japonicum* and *S. haematobium* adapted from the web site DPDx, which is developed by CDC's Division of Parasitic Diseases (DPD).

**Fig. (2):**
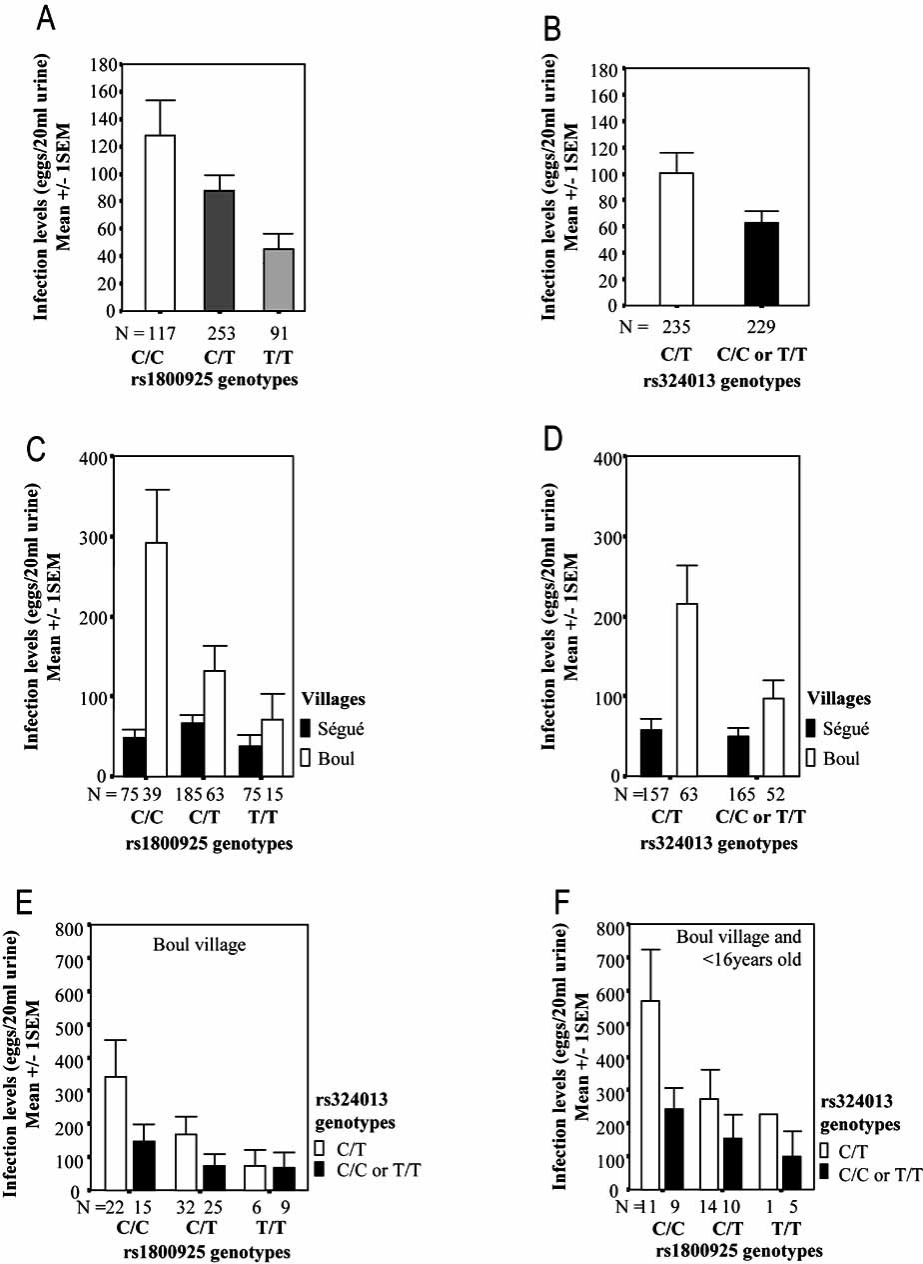
A: rs1800925T is associated with protection against infection by *Schistosoma haematobium* in the whole Malian study population. Infection levels were quantified by counting eggs in urine. Individual infection levels are the arithmetic mean egg counts on three to seven samples. To ensure quality control of the egg count, 10% of the filters were randomly selected and recounted by another microscopist. B: rs324013C/T is associated with high levels of infection caused by *Schistosoma haematobium* in the whole Malian study population. C: rs1800925T is stronger associated with protection against infection by *Schistosoma haematobium*, when taking into account only the most infected village, Boul. D: rs324013C/T is stronger associated with high levels of infection caused by *Schistosoma haematobium*, when considering only the most infected village, Boul. E: rs1800925 and rs324013 have additive effects on the regulation of infection levels caused by *Schistosoma haematobium*, when considering only the most infected village, Boul. F: rs1800925 and rs324013 have better additive effects on the regulation of infection levels caused by *Schistosoma haematobium*, when taking into account only the population younger than 16 years old, and coming from the most infected village, Boul.

**Fig. (3):**
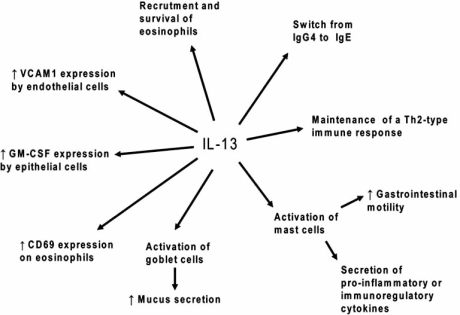
Multiple roles of the IL-13 in the protection against infection by *Schistosoma.*

**Fig. (4):**
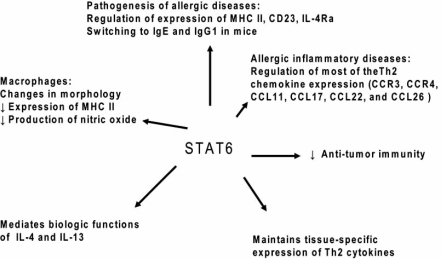
Different roles of STAT6 in the protection against infection by *Schistosoma* and in the regulation of the gene expression in the Th2 locus.
